# 393. Comparison of COVID-19 Associated Complications in Children during the First and Second Omicron Waves in Taiwan

**DOI:** 10.1093/ofid/ofad500.463

**Published:** 2023-11-27

**Authors:** Jiun-An Chen, Huan-Cheng Lai, Yu-Lung Hsu, Hsiao-Chuan Lin, Chien-Heng Lin, Hsiu-Mei Wei, Yan-Yi Low, Yu-Ting Chiu, Kao-Pin Hwang

**Affiliations:** China Medical University Children’s Hospital, China Medical University, Taichung, Taiwan, Taichung, Taichung, Taiwan; China Medical University Children’s Hospital, China Medical University, Taichung, Taiwan, Taichung, Taichung, Taiwan; China Medical University Children’s Hospital, China Medical University, Taichung, Taiwan, Taichung, Taichung, Taiwan; Division of Pediatric Infectious Diseases, China Medical University Children’s Hospital, Taichung, Taiwan, Taichung, Taichung, Taiwan; China Medical University Children’s Hospital, China Medical University, Taichung, Taiwan, Taichung, Taichung, Taiwan; China Medical University Children’s Hospital, China Medical University, Taichung, Taiwan, Taichung, Taichung, Taiwan; China Medical University Children’s Hospital, China Medical University, Taichung, Taiwan, Taichung, Taichung, Taiwan; China Medical University Children’s Hospital, China Medical University, Taichung, Taiwan, Taichung, Taichung, Taiwan; Division of Pediatric Infectious Diseases, China Medical University Children’s Hospital, Taichung, Taichung, Taiwan

## Abstract

**Background:**

In early 2022, the Omicron variant of the severe acute respiratory syndrome coronavirus 2 (SARS-CoV-2) emerged in Taiwan and caused two waves of COVID-19 outbreaks throughout the year. The emergence of the Omicron variant in Taiwan has led to an increased incidence of COVID-19 associated complications in children. This study aims to compare COVID-19 associated complications in children admitted during the peak of the first wave (late May, 2022, BA.2 predominance) and the second wave (early October, 2022, BA.5 predominance) of the Omicron-driven outbreaks in Taiwan.

**Methods:**

This retrospective, single-center, observational study was conducted at China Medical University Children's Hospital (CMUCH), a tertiary hospital in central Taiwan. The study population included children under 18 years of age who were admitted with a confirmed SARS-CoV-2 infection between May 1 and October 31, 2022. Medical records of these children were collected. The study period was divided into two periods based on the peak of the outbreak: May 1 to June 30, 2022 and September 1 to October 31, 2022. During these periods, the COVID-19 associated complications of hospitalized children were compared.

**Results:**

A total of 228 pediatric patients with SARS-CoV-2 infection were included, with 145 (63.6%) from the first wave and 83 (36.4%) from the second wave. The median age (IQR) was similar between the two waves [18 (6-43) months vs. 16 (5-62) months, *p* = 0.796]. The proportion of patients with underlying comorbidities was similar between the two waves (7.6% vs. 12.1%, *p* = 0.262). Patients in the second wave had a lower ICU admission rate (16.6% vs. 9.6%, *p* = 0.148). The proportion of patients with bacterial coinfection was higher in the second wave (15.9% vs. 24.1%, *p* = 0.126). The proportion of patients with febrile convulsion (13.8% vs. 16.9%, *p* = 0.531), myositis (5.5% vs. 2.4%, *p* = 0.334), and croup (11.0% vs. 16.9%, *p* = 0.148) were similar between the two waves.

**Conclusion:**

There was no significant difference in the frequency of admission for COVID-19 associated complications between the BA.2 and BA.5 predominance periods. However, it was observed that children admitted during the second wave had a lower rate of ICU admission and a higher rate of bacterial coinfection.

**Disclosures:**

**All Authors**: No reported disclosures

